# The diagnostic performance of magnetic resonance imaging for differentiating the nature of cardiac masses: A systematic review protocol

**DOI:** 10.1097/MD.0000000000018717

**Published:** 2020-01-10

**Authors:** Jin-Rong Ni, Yuan Hu, Li-Ping Shao, Bing Song, Yuan-Min Li, Jun-Qiang Lei

**Affiliations:** a The First Hospital (First Clinical Medical School) of Lanzhou University; b Department of Cardiovascular surgery, First Hospital of Lanzhou University; c Department of Ultrasound Medicine, Gansu Provincial Hospital; d Department of Radiology, First Hospital of Lanzhou University; e Intelligent Imaging Medical Engineering Research Center of Gansu Province; f Precision Image and Collaborative Innovation International Scientific and Technological Cooperation Base of Gansu Province, Lanzhou, China.

**Keywords:** cardiac, diagnosis, magnetic resonance, neoplasm, systematic review, tumor

## Abstract

**Background:**

Cardiac masses are rare, but lead to high risk of stroke and death. Because of the different treatment methods, it is significant for clinicians to differentiate the nature of masses. Cardiac magnetic resonance (CMR) imaging has high intrinsic soft-tissue contrast and high spatial and temporal resolution and can provide evidence for differential diagnosis of cardiac masses. However, there is no evidence-based conclusion as to its accuracy. Therefore, the purpose of our study is to perform a systematic review on this issue and provide useful information for clinical diagnosis and treatment.

**Methods:**

We will perform a systematic search in EMBASE, Cochrane Library, PubMed and Web of Science for diagnostic studies using CMR to detect cardiac masses from inception to October, 2019. Two authors will independently screen titles and abstracts for relevance, review full texts for inclusion and conduct detail data extraction. The methodological quality will be assessed using the QUADAS-2 tool. If pooling is possible, we will use bivariate model for diagnostic meta-analysis to estimate summary sensitivity, specificity, positive likelihood ratio, negative likelihood ratio, and diagnostic odds ratio of CMR, as well as different sequences of CMR. Estimates of sensitivity and specificity from each study will be plotted in summary receive operating curve space and forest plots will be constructed for visual examination of variation in test accuracy. If enough studies are available, we will conduct sensitivity analysis and subgroup analysis.

**Results:**

The results of this systematic review and meta-analysis will be published in a peer-reviewed journal.

**Conclusion:**

To our knowledge, this will be the first systematic review on the accuracy of CMR in the differential diagnosis of cardiac masses. This study will provide evidence and data to form a comprehensive understanding of the clinical value of CMR for cardiac masses patients.

**Ethics and dissemination:**

Ethics approval and patient consent are not required, as this study is a systematic review.

**PROSPERO registration number:**

CRD42019137800.

## Abstract 2

Supplemental Digital Content is available in the text

## 1 Introduction

Cardiac masses include tumors and thrombus. The estimated morbidity of cardiac tumor is 0.02% to 2.3% at autopsy and 0.15% at echocardiography.^[1]^ However, the incidence of thrombosis is high, especially in patients with atrial fibrillation and left ventricular systolic dysfunction, with the incidence of 3% to 25%^[2]^ and 2% to 50%^[3]^ respectively. Cardiac masses are potentially high-risk sources of embolism and sudden death.^[4]^ Differentiating the nature of cardiac masses (neoplastic or non-neoplastic, benign or malignant, primary or secondary) is particularly important because of the different treatment options.^[5]^

Cardiac magnetic resonance (CMR) imaging provides high spatial and temporal resolution, superior tissue characteristics, and large field of view that allows visualization of the cardiac structures in the absence of radiation exposure and is the gold standard in the diagnosis of many heart diseases.^[6,7]^ Combined evaluation of location, morphology, composition, and perfusion makes CMR a useful tool in the assessment of cardiac masses.^[8,9]^

However, studies about the accuracy of CMR for differential diagnosis of cardiac masses are scarce and have relatively small sample sizes.^[10,11]^ An ideal evidence system should integrate and evaluate all important research evidence related to specific clinical problems.^[12]^ A meta-analysis of diagnostic tests represents a powerful tool to summarize findings in the literature by considering and enabling analysis of differences between studies.^[13]^ Therefore, the present study is designed to synthesize currently available evidences to evaluate the value of CMR for differential diagnosis of cardiac masses.

## 2 Methods

We will adhere to the standers of the preferred reporting items for systematic reviews and meta-analyses (PRISMA) statement.^[14]^ The content of this protocol will follow the preferred reporting items for systematic review and meta-analysis protocols (PRISMA-P) recommendations.^[15]^ The protocol has been registered in PROSPERO, which is an International Prospective Register of Systematic Reviews. The registration number is CRD42019137800 (http://www.crd.york.ac.uk/PROSPERO/).

The system review does not require ethical approval or informed consent. There will be no direct contact with individual patients and only published data will be included in the review.

### 2.1 Types of studies

We will include all case-controlled studies or cross-sectional studies reporting the diagnostic accuracy of CMR for the differential diagnosis of cardiac masses. The histopathology and/or follow-up will be used as the reference standard. Studies in which sensitivity and specificity were reported or can be calculated will be included in the systematic review, but the meta-analysis of sensitivity and specificity estimates will be excluded.

#### 2.1.1 Participants

We will include studies of patients with suspected cardiac masses without age restriction.

#### 2.1.2 Index tests

We will include only studies of CMR for the evaluation of suspected cardiac masses. The scanning mode and imaging sequence of CMR are not regulated.

#### 2.1.3 Reference standards

The reference standards include biopsy, pathological results, autopsy, and/or follow-up.

#### 2.1.4 Exclusion criterion

The following studies will be excluded. Research not published in English; case reports, reviews, abstracts, or conference papers; unable to extract relevant data; sample size is <10.

### 2.2 Data sources and search strategy

We will perform a systematic search in EMBASE, Cochrane Library, PubMed, and Web of Science for relevant literatures from inception to June, 2019. Mesh words will be combined with free words, and the search strategies will be developed and adapted for each database (Supplementary 1). We will also review the references of included studies and other systematic reviews and meta-analysis to obtain a comprehensive list of included studies.

### 2.3 Citation management and screening

Citations will be imported and duplicates will be removed using EndNote X9 software (Thomson Reuters, Toronto, Ontario, Canada). Initially, 2 authors (JRN and YH) will independently screen the titles and abstracts and will eliminate those that do not meet the screening criteria. Next, 2 same authors (JRN and YH) will independently review the full text of the remaining studies to determine the eligible studies for suitability. Disagreements will be resolved by consensus. If consensus could not be reached, arbitration will be conducted by a third reviewer (LPS).

### 2.4 Data extraction

Using Microsoft Excel 2016 (Microsoft Corp, Redmond, WA, www.microsoft.com) to produce a standardized extraction form. The following data will be extracted independently by 2 authors (JRN and YH): name of the first author, publication year, country of study, study design, study sample size, mean age, male ratio, brand and model of CMR equipment, Imaging sequence of CMR, and the number of true-positive (TP), false-negative (FN), true-negative (TN), and false-positive (FP) observations. If such data were not provided by the trial authors, we will calculate the number of TP, TN, FP, and FN from the summary estimates of sensitivity and specificity of the index test or research object data lists. In some included studies, multiple data sets may need to be extracted for neoplastic/non-neoplastic, benign/malignant, primary/secondary, or different CMR sequences. For studies in which only a subgroup of patients were included in the review, we will extract, analyze, and present data for this subgroup only. Extracted data was cross-checked and disagreements were resolved via discussion or referral to a third reviewer (BS).

### 2.5 Quality assessment

The Quality Assessment of Diagnostic Accuracy Studies QUADAS-2 tool was used to assess the risk of bias and clinical applicability concerns of included studies as per the Cochrane Collaboration recommendation.^[16,17]^ Two authors (JRN and YH) will independently collect the information needed to assess the methodological quality of each included study. According to the Cochrane DTA Working Group's recommendation, the summary score will not be calculated because this obscures the importance of individual quality and can lead to inaccurate conclusion.^[18]^ Any differences will be resolved through discussion or with the help of a third author (YML).

### 2.6 Statistical analysis and data synthesis

The Review Manager (RevMan Version.5.3) software (Nordic Cochrane Centre, Cochrane Collaboration, Copenhagen, Denmark) will be used to document the descriptive analyses. If studies show enough clinical homogeneity or if these studies are conducted in the same or comparable context, we will synthesize the data. We will enter the data for the 2 × 2 tables into Stata/SE version 15.1 (Stata Corp, College Station, TX) and use the bivariate model for diagnostic meta-analysis to obtain pooled estimates of sensitivity, specificity, positive likelihood ratio (PLR), negative likelihood ratio (NLR), and diagnostic odds ratio (DOR) of CMR.^[19]^ In order to assess the variability between studies intuitively, we will display the results in forest plot after drawing sensitivity and specificity estimates and 95% confidence intervals (CIs) and draw receiver operating characteristic (ROC) curves.

### 2.7 Assessment of heterogeneity

To examine heterogeneity, we will visually inspect forest plots of each study sensitivities and specificities as well as ROC curves related to the individual study results. The correlation coefficient between the logarithm of sensitivity and logarithm of one minus specificity (i.e., the false-positive rate) will be calculated to test whether the threshold effect was one of the sources of heterogeneity.^[20]^ Statistical heterogeneity among studies will be explored using the *I*^2^ statistic. Significant heterogeneity is defined as *I*^2^ > 50%.

### 2.8 Assessment of publication biases

If a sufficient number of studies are included, we will evaluate publication bias through Deek funnel plots. Publication bias should be carefully interpreted because of its lack of statistical power and the lack of agreement on appropriate methods for detecting publication bias in diagnostic test accuracy assessments.

### 2.9 Sensitivity and subgroup analysis

After excluding studies with high bias risk or potential applicability doubts, sensitivity analyses will be conducted to determine the stability of the meta-analyses. Only when enough research is available can we conduct subgroup analysis to explore the sources of potential heterogeneity in sensitivity and specificity. Subgroup analysis may be performed as following categories: CMR imaging technology or sequence and CMR equipment field intensity.

## 3 Discussion

With the high intrinsic soft-tissue contrast and high spatial and temporal resolution, CMR allows for the assessment of cardiac masses. However, what is the diagnostic value of CMR for differential diagnosis of cardiac masses? Which imaging technique or sequence is the best choice for clinicians? To our knowledge, this review will be the first systematic review focusing on this issue. This systematic review will provide evidence to evaluate the clinical value of CMR in differential diagnosis of cardiac masses.

We acknowledge several limitations will present in this study. First, the incidence of cardiac masses is low, which may lead to a smaller sample size. Second, the quality of the imaging equipment and the ability of the imaging physician may skew the accuracy of the diagnosis. Notwithstanding its limitation, we hope to provide effective information for clinicians to figure out the diagnostic accuracy of CMR imaging methods and to recommend the optimal CMR technology or sequence for differential diagnosis of cardiac masses.

## Author contributions

JRN, YML, and JQL conceived the idea for this study; JRN and JQL designed the meta-analysis; JRN, YH, LPS, and BS provided statistical advice and input; JRN and YML drafted the protocol; YML and JQL reviewed the protocol and provided critical feedback.

## Supplementary Material

Supplemental Digital Content
