# Supplementary material for: The diagnostic performance of magnetic resonance imaging for differentiating the nature of cardiac masses: A systematic review protocol
Source: Medicine (Baltimore). 2020 Jan 10;99(2):e18717. doi: 10.1097/MD.0000000000018717 (PMC6959924; doi:10.1097/MD.0000000000018717)
Supplement: Supplemental Digital Content [file medi-99-e18717-s001.docx]

**Search strategy**

1. **Pubmed**

(((((((((((((((((((Heart Neoplasms[MeSH Terms]) OR Heart Neoplasm?[Title/Abstract]) OR Cardiac Neoplasm?[Title/Abstract]) OR Cardiac tumor?[Title/Abstract]) OR Heart tumor?[Title/Abstract]) OR Cardiac sarcoma?[Title/Abstract]) OR Heart sarcoma?[Title/Abstract]) OR Heart cancer？[Title/Abstract]) OR Cardiac cancer?[Title/Abstract]) OR Cardiac Carcinoma?[Title/Abstract]) OR Heart Carcinoma?[Title/Abstract]) OR cardiac myxoma[Title/Abstract]) OR cardiac mass[Title/Abstract]) OR cardiac masses[Title/Abstract]) OR heart mass[Title/Abstract]) OR heart masses[Title/Abstract]) OR Myocardial Tumor?[Title/Abstract]) OR Intracavitary Tumors of the Heart[Title/Abstract])) AND ((((((magnetic resonance imaging[MeSH Terms]) OR magnetic resonance imaging[Title/Abstract]) OR CMR[Title/Abstract]) OR MRI[Title/Abstract]) OR MR[Title/Abstract]) OR CMRI[Title/Abstract])

1. **EMBASE**

('heart tumor'/exp OR 'heart tumor':ab OR 'heart tumour':ab OR 'heart tumors':ab OR 'cardiac tumors':ab OR 'cardiac tumor':ab OR 'cardiac tumour':ab OR 'cardiac tumours':ab OR 'heart tumours':ab OR 'heart neoplasms':ab OR 'heart neoplasm':ab OR 'cardiac neoplasm':ab OR 'cardiac neoplasms':ab OR 'cardiac cancer':ab OR 'heart cancers':ab OR 'heart cancer':ab OR 'cardiac cancers':ab OR 'cardiac mass':ab OR 'cardiac masses':ab OR 'heart masses':ab OR 'heart mass':ab OR 'heart sarcoma':ab OR 'heart sarcomas':ab OR 'cardiac sarcomas':ab OR 'cardiac sarcoma':ab OR 'cardiac carcinoma':ab OR 'cardiac carcinomas':ab OR 'heart carcinomas':ab OR 'heart carcinoma':ab OR 'heart myxoma':ab OR 'heart myxomas':ab OR 'cardiac myxomas':ab OR 'cardiac myxoma':ab OR 'myocardial tumor':ab OR 'myocardial tumors':ab) AND ('cardiovascular magnetic resonance'/exp OR 'cardiovascular magnetic resonance':ab OR 'magnetic resonance imaging':ab OR 'cmr':ab OR 'mr':ab OR 'mri':ab OR 'cmri':ab)

**3. Cochrane Library**

#1 MeSH descriptor: [Heart Neoplasms] explode all trees 16

#2 (heart cancer?):ti,ab,kw (Word variations have been searched) 5870

#3 (cardiac cancer?):ti,ab,kw (Word variations have been searched) 2519

#4 (cardiac tumor?):ti,ab,kw (Word variations have been searched) 1545

#5 (heart tumor?):ti,ab,kw (Word variations have been searched) 2962

#6 (heart neoplasm?):ti,ab,kw (Word variations have been searched) 2346

#7 (cardiac neoplasm?):ti,ab,kw (Word variations have been searched) 1142

#8 (heart carcinoma?):ti,ab,kw (Word variations have been searched) 1058

#9 (cardiac carcinoma?):ti,ab,kw (Word variations have been searched) 491

#10 (cardiac mass):ti,ab,kw (Word variations have been searched) 2940

#11 (cardiac masses):ti,ab,kw (Word variations have been searched) 2938

#12 (heart masses):ti,ab,kw (Word variations have been searched) 8343

#13 (heart mass):ti,ab,kw (Word variations have been searched) 8349

#14 (cardiac myxoma?):ti,ab,kw (Word variations have been searched) 22

#15 (heart myxoma?):ti,ab,kw (Word variations have been searched) 26

#16 (Myocardial Tumor?):ti,ab,kw (Word variations have been searched) 524

#17 (Intracavitary Tumors of the Heart):ti,ab,kw (Word variations have been searched) 9

#18 #1 or #2 or #3 or #4 or #5 or #6 or #7 or #8 or #9 or #10 or #11 or #12 or #13 or #14 or #15 or #16 or #17 18619

#19 MeSH descriptor: [Magnetic Resonance Imaging] explode all trees 7673

#20 ("magnetic resonance imaging"):ti,ab,kw (Word variations have been searched) 21881

#21 (MRI):ti,ab,kw (Word variations have been searched) 20852

#22 (MR):ti,ab,kw (Word variations have been searched) 5704

#23 (CMR):ti,ab,kw (Word variations have been searched) 847

#24 #19 or #20 or #21 or #22 or #23 33119

#25 #18 and #24 1069

**4. Web of Science**

#1 TS=("Heart Neoplasms" OR "Heart Neoplasm？'" OR "Cardiac Neoplasm？" OR "Cardiac tumor?" OR "Heart tumor?" OR "Cardiac sarcoma?" OR "Heart sarcoma?" OR "Heart cancer?" OR "Cardiac cancer?" OR "Cardiac Carcinoma?" OR "Heart Carcinoma?" OR "cardiac myxoma" OR "cardiac mass" OR "heart myxoma" OR "cardiac masses" OR "heart mass" OR "heart masses" OR "Myocardial Tumor?" OR "Intracavitary Tumors of the Heart")

#2 TS=("magnetic resonance imaging" OR "cardiovascular magnetic resonance" OR MRI OR CMR OR MR OR CMRI )

#3 #1 and #2
